# Off-clamp robotic-assisted partial nephrectomy

**DOI:** 10.1590/S1677-5538.IBJU.2015.0386

**Published:** 2016

**Authors:** Alec J. Wright, Aaron M. Potretzke, Jeffrey Larson, Robert S. Figenshau

**Affiliations:** 1 Division of Urologic Surgery, Washington University, St. Louis, Missouri, USA

## INTRODUCTION

Robotic-assisted partial nephrectomy (RAPN) is an effective option for the management of small renal masses. Off-clamp RAPN has emerged as a method to minimize renal ischemic injury and improve renal functional outcomes. In this video we demonstrate our technique for off-clamp RAPN in a patient with a complex 3.6cm hilar tumor.

## MATERIALS AND METHODS

We performed a retrospective review of 187 patients who received off-clamp RAPN between August 2007 and March 2015. Patient demographic information, operative information, perioperative outcomes, and renal functional outcomes were evaluated. The Chronic Kidney Disease Epidemiology Collaboration formula was used to calculate eGFR.

## RESULTS

The 187 patient cohort had a mean (±standard deviation) age of 58.8±11.8 years and a mean BMI of 32.2±8.3kg/m^2^. The mean clinical tumor size was 2.9±1.6cm with a mean nephrometry score of 6.9±2.1. There was a mean operative time of 169±61 minutes and a mean estimated blood loss of 242±299mL. Positive surgical margins occurred in 2 cases (1.1%). Average hospital length of stay was 2±1.3 days, and there were 14 (7.5%) postoperative complications. Mean percent decline of eGFR was 6.8±21.4%. The average follow-up was 454 days (15-2195 days) with 1 recurrence (0.53%) occurring at day 596.

## CONCLUSION

Off-clamp RAPN, which eliminates warm ischemic time, is both safe and effective in the treatment of small renal masses when performed by an experienced surgeon. We have found this technique is appropriate for most tumors amenable to minimally invasive partial nephrectomy.

## ARTICLE INFO

**Available at:** **www.intbrazjurol.com.br/video-section/wright_1044_1045/**

**Int Braz J urol. 2016; 42 (video #10): 1044-5**

